# The NAD^+^ Responsive Transcription Factor ERM-BP Functions Downstream of Cellular Aggregation and Is an Early Regulator of Development and Heat Shock Response in *Entamoeba*

**DOI:** 10.3389/fcimb.2020.00363

**Published:** 2020-07-17

**Authors:** Dipak Manna, Daniela Lozano-Amado, Gretchen Ehrenkaufer, Upinder Singh

**Affiliations:** ^1^Division of Infectious Diseases, Stanford University School of Medicine, Stanford, CA, United States; ^2^Department of Microbiology and Immunology, Stanford University School of Medicine, Stanford, CA, United States

**Keywords:** *Entamoeba*, transcription factor, encystation, heat-shock, multinucleated giant cells

## Abstract

*Entamoeba histolytica* is a protozoan parasite and a major cause of dysentery and diarrheal disease in developing countries. Disease transmission from one host to another occurs via cysts which can survive in environmental extremes and are transmitted through contaminated food and water. Recent studies in our lab identified a novel transcription factor, Encystation Regulatory Motif- Binding Protein (ERM-BP), which is responsive to NAD^+^ and has an important role in encystation. The key residues important for ERM-BP function were demonstrated *in vitro* using recombinant protein. In this study we demonstrate the *in vivo* functional consequences of mutations in key domains and their impact on *Entamoeba* encystation. Our results show that mutations in the DNA binding domain (ERM-BP-DBM) and in the nicotinamidase domain (ERM-BP-C198A) lead to protein mis-localization in both trophozoites and cysts and significantly reduce encystation efficiency. Additionally, we showed that silencing of ERM-BP significantly decreased the size and number of multi-nucleated giant cells (MGC) that form during encystation, indicating that ERM-BP functions upstream of the cellular aggregation that precedes stage conversion. Dissection of epistatic interactions between ERM-BP and a second encystation-related transcription factor, NF-Y revealed that ERM-BP is upstream of NF-Y in controlling the developmental cascade and appears to be one of the earliest regulators of development identified to date in *Entamoeba*. We also demonstrated that ERM-BP is upregulated during heat stress in *Entamoeba*, another condition which increases intracellular NAD^+^ levels and that overexpression of ERM-BP makes *E. histolytica* and *E. invadens* parasites more resistant to heat stress. Overexpression of ERM-BP in *E. histolytica* also induced the formation of cyst-like quadrinucleated cells and formation of MGCs. Overall, our work has identified an important role of ERM-BP in *Entamoeba* stress response and links an NAD^+^-responsive transcription factor to both development and heat shock response. Characterization of stress and developmental cascades are important avenues to investigate for *Entamoeba*, an important human parasitic pathogen.

## Introduction

The protozoan parasite *Entamoeba histolytica* causes an estimated 50 million cases of invasive disease annually and is the second leading parasitic cause of death worldwide (Haque et al., 2003; Lozano et al., 2012). The life cycle of the parasite involves inter-conversion between trophozoites, a stage which invades tissue and causes clinical disease and cysts, a stage which transmits disease in contaminated food or water (McConnachie, 1969). However, the molecular controls of the developmental life cycle of this parasite are poorly studied, and the triggers that initiate stage conversion are not well-delineated. Most developmental studies have been done in a closely related reptilian parasite, *E. invadens*, which can be encysted *in vitro* using glucose depletion and osmotic stress (Avron et al., 1986) and excysted from cysts to trophozoites using media supplemented with glucose, bile salt, sodium bicarbonate and serum (Mitra and Krishna Murti, 1978). Using this model of *Entamoeba* development, a number of triggers of encystation including catecholamine, gal-terminated ligands, cyclic AMP (cAMP), cholesteryl sulfate, NAD^+^, Ca^2+^ signaling, and phospholipase-D (PLD) have been identified (Chayen et al., 1985; Cho and Eichinger, 1998; Eichinger, 2001; Makioka et al., 2001; Coppi et al., 2002; Frederick and Eichinger, 2004; Ehrenkaufer et al., 2013; Martinez-Higuera et al., 2015; Mi-ichi et al., 2015; Manna et al., 2018; Manna and Singh, 2019). Furthermore, a number of molecules, e.g., galactose, N-acetylglucosamine, and short chain fatty acids, have been shown to inhibit encystation (Coppi and Eichinger, 1999; Byers et al., 2005). It has also recently been noted that multinucleated giant cells (MGC), which originate from cell aggregates due to fusion of multiple trophozoites, develop during encystation (Krishnan and Ghosh, 2018) indicating that *Entamoeba* encystation and MGC formation are induced by similar physiological conditions and key regulators, and may share similar control pathways.

In the human pathogen *E. histolytica, in vitro* regulated encystation has not been accomplished to date. Reports of cyst-like structures from *E. histolytica* exposed to stress conditions have been reported, but these are not mature cysts as they lack a thick chitinous cyst walls and there are no evidence that these can excyst to trophozoites; instead most likely, these are parasites attempting to encyst (Barron-Gonzalez et al., 2008; Aguilar-Diaz et al., 2010). Using parasites isolated from patients and maintained in xenic conditions, low-level, continuous encystation and excystation has been noted (Ehrenkaufer et al., 2007). In these conditions, mature quadrinucleated cysts and thick cyst walls were produced, but with low efficiency. Heat shock stress has been noted to have some overlap with encystation as genes involved in cyst wall formation (e.g., Chitinase and Jacob) are noted to be upregulated in both conditions (Field et al., 2000).

Three transcription factors with important roles in *Entamoeba* encystation have been identified. Two of these transcription factors - a developmentally regulated Myb protein (drMyb), which binds a hexanucleotide promoter motif CCCCCC, and an Encystation Regulatory Motif-Binding Protein (ERM-BP), which binds a hepta-nucleotide promoter motif, CAACAAA-appear at early time point (24 h) of encystation (Ehrenkaufer et al., 2009; Manna et al., 2018). The third transcription factor, a nuclear factor complex (NF-Y) is composed of three subunits NF-YA, NF-YB, and NF-YC, which bind to a pentanucleotide motif CCAAT and appears at a later time point of encystation (48 h) (Manna and Singh, 2019). Silencing of ERM-BP results in a significantly reduced encystation efficiency and abnormal cysts with defective cyst walls which have reduced viability under excystation conditions (Manna et al., 2018). The function of ERM-BP is regulated by direct binding of the metabolic cofactor NAD^+^; binding to NAD^+^ changes protein conformation and facilitates ERM-BP binding to the DNA motif. ERM-BP is also capable of nicotinamide catalysis. Biochemical studies using ERM-BP recombinant protein revealed key residues and domains important for NAD^+^ binding, DNA binding, and nicotinamide catalysis. A five amino acid cluster at the N-terminus of ERM-BP (S_A_R_L_TKR) except “A” and “L” as shown in subscripts is a DNA-binding domain which is crucial for DNA binding and a cysteine residue at the C-terminal nicotinamidase domain (C198) is crucial for NAD^+^ binding (Manna et al., 2018).

In this study we evaluated the functions of ERM-BP domains *in vivo* by overexpressing mutant versions of the protein in *Entamoeba* parasites and assessing phenotypic outcomes. Our results show that mutations in the DNA binding domain (ERM-BP-DBM) and a single amino acid change in the nicotinamidase domain (ERM-BP-C198A) lead to mis-localization of the mutant protein in both trophozoites and cysts and significantly reduce encystation efficiency. Our study also revealed a role for ERM-BP in formation of multi-nucleated giant cells (MGC) during encystation. We showed that silencing of ERM-BP significantly decreased the number of giant cells formed indicating that encystation and giant cell formation may share similar signaling pathway which is affected by loss of function of ERM-BP. Furthermore, we showed that in heat stress, another condition in which NAD^+^ levels increase, ERM-BP is upregulated in both *E. invadens* and in *E. histolytica*. Nuclear extracts from heat stressed parasites specifically bind to ERM, and overexpression of ERM-BP makes parasites more resistant to heat stress. Overexpression of ERM-BP in *E. histolytica* produces quadrinucleate cyst-like structures, and multinucleated giant cells also observed due to heat stress, supporting the concept that heat-stress response and encystation are related. Overall, our work identified an important role of ERM-BP which functions downstream of cellular aggregation and is an early regulator of development and heat shock response in *Entamoeba*.

## Materials and Methods

### Parasite Culture, Transfection, and Induction of Stage Conversion

*E. invadens* (strain IP-1) was axenically maintained as described earlier (Clark and Diamond, 2002). To make stable transgenic cell lines, parasites were transfected with plasmid DNA by electroporation (Ehrenkaufer and Singh, 2012). Stable cell lines were maintained at G418 concentration of 80 μg/mL unless otherwise stated. To induce encystation, *E. invadens* trophozoites were incubated in 47% LYI-LG (supplemented with 7% adult bovine serum). Encystation efficiency was determined by counting the number of cells before and after sarkosyl treatment. Data are represented as mean with standard deviation and the *t*-test was performed from well-distributed data set (*n* = 3) of each cell line. Trophozoites of *E. histolytica* strain HM-1:IMSS were grown under axenic conditions in TYI-S-33 medium (Diamond et al., 1978). EhERM-BP (EHI_146360) cell line constitutively overexpressing N-terminally Myc-tagged EhERM-BP was made by using the plasmid pKT-3M as a backbone and maintained at G418 concentration of 12 μg/mL.

### Immunostaining

*E. invadens* trophozoites and cysts expressing myc-tagged WT and mutant versions of ERM-BP were fixed with acetone/methanol (1:1) and permeabilized with 0.1% Triton X-100 as described earlier (Manna et al., 2018). Cells were incubated with 3% bovine serum albumin (BSA) for blocking followed by mouse monoclonal anti-myc antibody (1:500, Cell signaling). Heat-shocked and control *E. histolytica* trophozoites expressing myc-tagged ERM-BP fixed with acetone/methanol (1:1) and permeabilized with 0.1% Triton X-100. Slides were prepared using Vectashield mounting medium with DAPI (Vector Laboratories, Inc) and visualized using a Leica CTR6000 microscope, using a BD CARVII confocal unit. Images were analyzed using Leica LAS-AF software.

### Live Cell Imaging

*E. invadens* cells were encysted for 72 h and stained with cell permeable Cyto11 (stains DNA) and calcofluor white (stains cyst wall) in a 96-well plate. Cells were continuously visualized under 20 × objective using three channels (Bright Field, FITC, and DAPI) in a Leica CTR6000 microscope and time-lapse images were captured at 1 s intervals for the indicated time periods. Images were analyzed using Leica LAS-AF software. The movie represents all the channels as merged and 3 frames per second (.mov file).

### Electrophoretic Mobility Shift Assays (EMSA)

EMSA was performed as previously described (Pearson et al., 2013). The oligonucleotides used in EMSA are listed in Supplementary Table 1. Each motif had an additional 12-nt at 5′ and 8-nt at 3′, which creates a 5′-overhang after annealing and was utilized for radiolabeling using Klenow (Hackney et al., 2007). In brief, complementary overlapping ERM-probes were annealed and labeled using [^32^P] α-ATP and Klenow fragment (Invitrogen). Binding reaction was set in a total volume of 20 μl, which included 2 μl 10 × EMSA binding buffer (10 mM Tris-HCl, pH 7.9, 50 mM NaCl, 1 mM EDTA, 3% glycerol, 0.05% milk powder, and 0.05 mg of bromophenol blue), 5 μg of nuclear extract form control and heat-shocked trophozoites, 2 μg of poly (dI-dC), and 50 fmol of labeled probe. The binding reaction mixes were loaded onto a 9% non-denaturing polyacrylamide gel and run for 3 h. The gel was fixed, dried, and exposed to a phosphor screen. Gels were imaged using Personal Molecular Imager (PMI) system with Quantity One software, Bio-Rad.

### RNA Extraction and RT-PCR

Total RNA was extracted from trophozoites using TRIzol method (Life Technologies). RNA was subjected to DNase treatment (DNase kit; Invitrogen) and reverse transcribed using oligo (dT) primers (Invitrogen). The resultant cDNA (3 μl) was used in subsequent PCRs (25 μl total volume). The number of PCR cycles was set to 30, and 10 μl of PCR products was run on a 1.5% agarose gel. The negative control (minus reverse transcriptase [RT]) was split away before the addition of Superscript RT (Invitrogen) and otherwise treated like the other samples. The primers used in RT-PCR are listed in Supplementary Table 1.

### Plasmid Construction

To overexpress the protein in *Entamoeba* the full-length coding region of ERM-BP gene (EIN_083100) was cloned into the AvrII and SacII sites in the pEi-CKII-myc plasmid as previously described (Manna et al., 2014, 2018). For the mutants, PCR was done from pGEX-2T1 clones (ERM-BP-D12A, ERM-BP-DBM, ERM-BP-K150A, and ERM-BP-C198A) as described earlier and cloned in the pEi-CKII-myc backbone plasmid at AvrII and SacII sites. For the cloning of *E. histolytica* homolog of ERM-BP (EHI_146360) the full-length coding region of EHI_146360 was cloned into pKT-3M backbone as described earlier (Zhang et al., 2008). The primers used in cloning are listed in Supplementary Table 1. The constructs were confirmed by sequencing before transfecting into *Entamoeba*.

### Measurement of Intracellular NAD^+^/NADH

Intracellular NAD^+^ and NADH were determined as per the manufacturer's protocol (NAD^+^/NADH Assay Kit, Cat No: ab65348, Abcam) and as described earlier (Manna et al., 2018). Briefly 2 × 10^6^ control or heat-shocked cells were lysed in NAD^+^/NADH extraction buffer by sonication (five pulses at 15 amp for 15 s). The lysate was centrifuged at 14,000 rpm and the supernatant containing NAD^+^/NADH was filtered through a 10 kDa spin column to get rid of enzymes, which may consume NADH rapidly. To detect the NADH in the sample, a decomposition step was performed by heating the samples at 60°C for 30 min; under this condition, all the NAD^+^ will be decomposed while NADH will be still intact. 100 μl reaction mix was prepared for each standard and samples were plated in duplicates in a clear bottom 96 well plate (Corning, Catalog # CLS3603). The plate was incubated at room temperature for 5 min to convert NAD^+^ to NADH followed by addition of 10 μl NADH developer into each well and incubated at room temperature for 2 h. OD was measured at 450 nm using a plate reader (BioTek Cytation3).

### Induction of Heat Stress and Viability Assay

For *E. histolytica*, heat shock was induced in a 42°C water bath for different time points (1, 2, 3, and 8 h). For *E. invadens*, heat shock was induced in a 37°C water bath for different time points (1, 2, 3, and 8 h). RNA expression by RT-PCR was performed at the time point as indicated. Viability assay was performed by Fluorescein diacetate (FDA) hydrolysis assay. Cells were pelleted and resuspended in PBS containing 10 μg/ml FDA. Cells were incubating at room temperature for 5 min followed by wash with 1 × PBS. The cells were then observed under a fluorescence microscope for the fluorescence produced by live cells.

### Statistical Analysis

Student's *t*-test was performed for all experiments where two conditions or genotypes were compared. A *p*-value of < 0.05 in each independent experiment was considered significant.

## Results

### Dominant Negative Effect of ERM-BP Mutants on Encystation Efficiency

Transcription factors play a crucial role in controlling different life cycle stages in many organisms. Recent studies in *Entamoeba* identified a novel developmentally regulated transcription factor ERM-BP which has important roles in the encystation process (Manna et al., 2018). Silencing of ERM-BP significantly reduces the encystation efficiency and produces ghost-like cysts, which fail to undergo excystation to trophozoites (Manna et al., 2018). Biochemical studies with recombinant proteins identified distinctive roles of its two domains, an N-terminal DNA-binding domain (DBD) and a C-terminal nicotinamidase domain (Manna et al., 2018). A single amino acid mutation at the Cysteine residue at position 198 to Alanine (C198A) in the nicotinamidase domain (ERM-BP-C198A) impairs both NAD^+^ and DNA binding activity. On the other hand, mutation in a five amino acid cluster (S_A_R_L_TKR) in the DNA-binding domain (ERM-BP-DBM) significantly affects DNA binding properties; however, this mutant can bind NAD^+^. Two other mutants, ERM-BP-D12A and ERM-BP-K150A, retain both NAD^+^ and DNA binding properties but have reduced enzymatic activity for conversion of nicotinamide to nicotinic acid (Manna et al., 2018).

**Figure 1:**
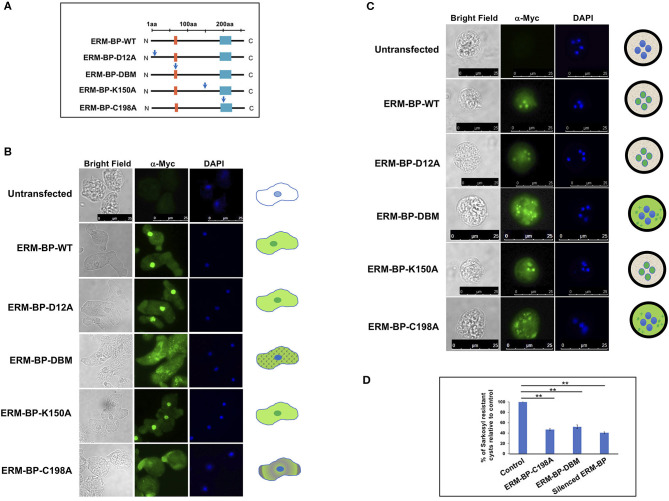
Functional characterization of ERM-BP-WT and mutants and dominant negative effects of C198A and ERM-BP-DBM. **(A)** Different constructs expressing WT and mutant versions of ERM-BP and with Myc-tagged at the N-terminal end are generated by changing each relevant residue into an Alanine as pointed by the blue arrows. Orange box at the N-terminus indicates location of the predicted DNA binding domain (S_A_R_L_TKR) and cyan-box at the C-terminus indicates the nicotinamidase domain which is important for NAD binding. The mutants are ERM-BP-D12A, ERM-BP-C198A, ERM-BP-K150A and a DNA binding mutant (ERM-BP-DBM) in which five amino acids cluster (S_A_R_L_TKR) except “A” and “L” as shown in subscripts in the orange-box were changed into Alanine. All the constructs were transfected into *E. invadens* and stable cell lines were generated. Immunostaining with α-myc antibody in **(B)** trophozoites, and **(C)** 24 h cysts were performed in ERM-BP-WT and mutant cells (Green). DNA was stained with DAPI (Blue). The pattern of localization in both trophozoites and cysts are shown in schematic on the right. Scale bars for both trophozoites and cysts are 25 μm. **(D)** Data represents the percentage of sarkosyl resistant cysts relative to control after 72 h of encystation. Data are mean ± s.d. (*n* = 3) Student's *t*-test; **P < 0.01.

The above studies were done with recombinant protein in the absence of other amebic cellular factors or proteins. In the current study, we characterized the function of the ERM-BP mutants in *E. invadens* parasites by overexpressing WT and mutant proteins and assessing the phenotypes of the parasites in development. All the mutants and WT-constructs were generated in an *E. invadens* expression vector under CK-promoter and myc-tagged at the N-terminus as described earlier (Ehrenkaufer and Singh, 2012). Figure 1A is a schematic of ERM-BP-WT and various mutants with the position of the mutation being indicated by the arrow. All the constructs were transfected into *E. invadens* and stable cell lines were generated. The expression of proteins was tested by western blot analysis using anti-myc antibody. To determine intracellular localization, immunostaining was performed with α-myc antibody in trophozoites and 24 h cysts. Overexpression of mutant ERM-BP-D12A and ERM-BP-K150A showed nuclear and cytoplasmic localization in trophozoites similar to ERM-BP-WT (Figure 1B); this is consistent with the *in vitro* results where recombinant ERM-BP-D12A and ERM-BP-K150A retain the ability to bind DNA. However, ERM-BP-C198A and ERM-BP-DBM showed only cytoplasmic localization in trophozoites. ERM-BP-DBM showed intense puncta like structure throughout the cytoplasm and ERM-BP-C198A show localization at the pseudopod (Figure 1B). A similar pattern of localization was seen in cysts where ERM-BP-D12A and ERM-BP-K150A localized to the nucleus and ERM-BP-C198A and ERM-BP-DBM mutants showed disintegrated localization in the cytoplasm (Figure 1C). Consistent with the altered localization in trophozoites and cysts, overexpression of both ERM-BP-C198A and ERM-BP-DBM mutants significantly reduced encystation efficiency, indicating that these mutants exerted dominant negative effects on the efficiency of stage conversion (Figure 1D).

### Silencing of ERM-BP Affects Formation of Multi-Nucleated Giant Cells

**Figure 2:**
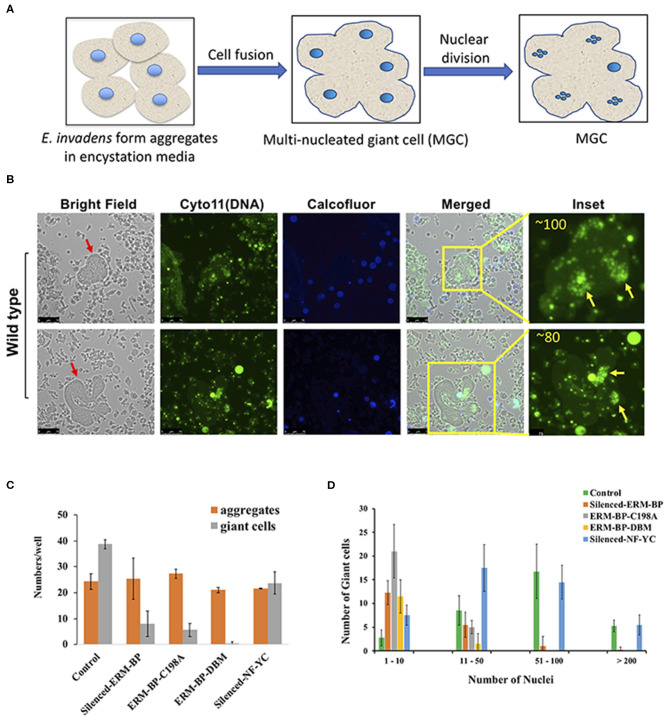
*E. invadens* form Multi-nucleated Giant Cells (MGC) during encystation and silencing of ERM-BP and mutants restrict the formation of MGC during encystation. **(A)** Schematic depicts the formation of giant cells during encystation. Amoeba form aggregates after inoculation into encystation media. Multiple cells can fuse together to make a giant cell. The polyploid entamoeba nucleus undergoes miotic division to make quadrinucleate haploid nuclei and remain as clustered. **(B)** Staining of 72 h encysted cells with cell permeable Cyto11 (in green for DNA) and calcofluor white (in blue for cyst wall) are shown in two representative fields. Red arrow indicates the giant cells. In the inset, nuclear staining observed at higher magnification and the number of nuclei in each giant cell is shown. Yellow arrows indicate the clusters of nuclei in those giant cells. Scale bars are 25 μm. **(C)** Control, silenced-ERM-BP, two other mutants ERM-BP-C198A, ERM-BP-DBM, and silenced-NF-YC were encysted in 96-well plates in six replicates per plate for 72 h. The number of aggregates and number of giant cells per well were represented. **(D)** The number of giant cells and their corresponding number of nuclei content (nuclei numbers: 1–10, 11–50, 51–100, and >200) are shown in control, silenced-ERM-BP, ERM-BP-C198A, ERM-BP-DBM, and silenced-NF-YC. Data are mean ± s.d. (*n* = 3).

Cell fusion can lead to formation of giant cells with multiple nuclei in many systems (Milde et al., 2015; Miron and Bosshardt, 2018). Formation of multi-nucleated giant cells (MGC) can increase in response to an infection, such as tuberculosis, herpes, HIV, or other foreign body (Anderson, 2000; Dargent et al., 2000; Brodbeck and Anderson, 2009; McClean and Tobin, 2016). Formation of MGC is also observed in the development of soil-dwelling amoeba *Dictyostelium discoideum* during macrocyst formation (Ishida et al., 2005) and in *E. invadens* during the encystation (Krishnan and Ghosh, 2018). In encystation media *Entamoeba* form aggregates and multiple cells go through cytofusion and transform into giant cells (Figure 2A). MGC nuclei are smaller compared to trophozoites suggesting that MGC underwent nuclear division similar to that seen in cysts (Krishnan and Ghosh, 2018) and meiotic genes were reported which expressed during encystation (Ehrenkaufer et al., 2013). These tiny nuclei inside the MGC are mostly clustered and can go through nuclear fusion to make a polyploid nucleus. The mechanisms and signaling responsible for MGC formation are not yet known.

Interestingly, we observed that the silencing of ERM-BP significantly restricts the formation of MGC compare to control parasites. The MGC were observed at a very low frequency in WT-cells (1 in 10^4^ cells) and the size of MGC increases with time of incubation of cells in encysting medium due to continuous fusions of cells. Similar to published data, our analysis identified increasing frequency and size of MGC with prolonged exposure in encystation conditions. By 72 h of encystation, in control cell lines the giant cells are very large and most of the giant cells contain over 50 nuclei (Figure 2B and Supplementary Movies 1, 2). Parasites in which ERM-BP is disrupted (silenced ERM-BP, ERM-BP-C198A, and ERM-BP-DBM) had decreased formation of MGC number and those MGC that were formed were significantly smaller (Figure 2C). However, there was no significant difference in the formation of cellular aggregates, suggesting that the MGC formation is downstream of aggregate formation and dependent on ERM-BP function. Silencing of another transcription factor, NF-YC, which appears later in encystation (48 h) showed a moderate effect on MGC formation (Figure 2C). In the MGC observed where ERM-BP function is disrupted (ERM-BP silenced, ERM-BP-C198A, and ERM-BP-DBM) the number of nuclei is predominantly between 1 and 10. On the contrary, the nuclei number in control parasites and parasites with silenced-NF-YC ranges between 51 and 100, and in few MGC it's even more than 200 (Figure 2D). Our results indicate that encystation pathway and formation of MGC may share similar signaling pathway(s); both formation of MGC and encystation are affected by ERM-BP function.

### NAD^+^ Increases With Heat Shock and Overexpression of ERM-BP Makes Parasites More Resistant to Heat Stress

**Figure 3:**
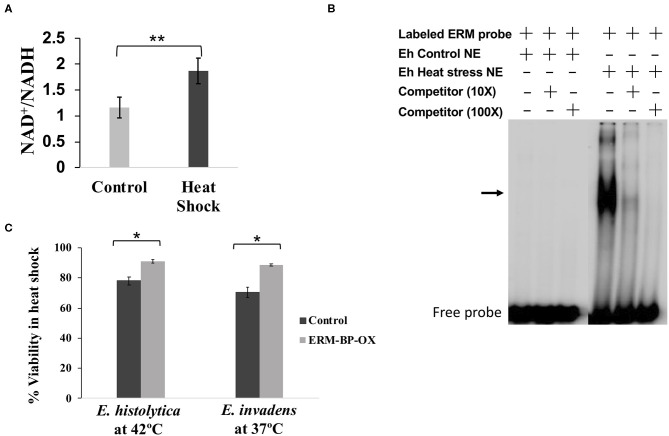
Intracellular NAD^+^/NADH elevated due to heat-stress, nuclear protein from heat shock parasites binds specifically to ERM and overexpression of ERM-BP makes parasites more resistant to heat stress. **(A)** Measurement of intracellular NAD^+^/NADH in heat-shock and untreated *E. histolytica*. Data are mean ±s.d. (*n* = 3) Student's *t*-test; **p < 0.01. **(B)** EMSA was performed using crude nuclear extracts from control and heat-stressed *E. histolytica* cells. EMSA results are shown in the presence and absence of different components marked as “+” and “–,” respectively. Radiolabeled ERM probe was used in each reaction. Unlabeled ERM probe at 10× and 100× was used as a specific competitor as indicated. The arrow indicates the major specific band in the gel shift assay; free probe is at the bottom. **(C)** Viability assay of control and overexpressed-ERM-BP cells from both *E. histolytica* and *E. invadens* were performed by FDA hydrolysis assay. Data are mean ± s.d. (*n* = 2) Student's *t*-test; *P < 0.05.

It has previously been shown that stress response important for encystation appears to overlap with that of with heat stress (Field et al., 2000). Thus, we looked to determine whether ERM-BP expression is only specific for encystation or also has correlation with other stress response. RT-PCR revealed that expression of ERM-BP is upregulated in heat stress in *E. histolytica* trophozoites (Supplementary Figure 1). However, expression of ERM-BP was not affected by H_2_O_2_ stress (data not shown). In order to determine if NAD^+^ levels changed during heat shock, we measured NAD^+^/NADH levels in control and heat-shocked *E. histolytica* trophozoites. Our results demonstrated that intracellular NAD^+^ levels goes up significantly in heat stressed parasites (Figure 3A). This level of increase is similar to what we noted in *E. invadens* encysting parasites (Manna et al., 2018). Additionally, we tested nuclear extracts from heat-shocked *E. histolytica* trophozoites, which showed specific binding to ERM compared to control parasites where no binding was noted (Figure 3B).

In order to define a phenotype with ERM-BP and heat shock response, we overexpressed ERM-BP in both *E. invadens* and *E. histolytica* and exposed the parasites to heat shock for different time points (0, 1, 2, 3, 8 h). For heat stress, *E. invadens* cells were incubated at 37°C and *E. histolytica* at 42°C and cellular viability was determined at different time points (Supplementary Figure 2). We observed that overexpression of ERM-BP makes both *E. invadens* and *E. histolytica* more resistant to heat stress after 8 h of incubation (Figure 3C). Thus, it appears that ERM-BP has a dual role in *E. invadens* – for regulating both encystation and heat shock response. In *E. histolytica*, ERM-BP is important in mediating heat shock stress response; encystation in *E. histolytica* cannot be assessed.

### Heat Stress and Overexpression of ERM-BP Induce the Formation of Quadrinucleated Cyst-Like Structures and MGC

**Figure 4:**
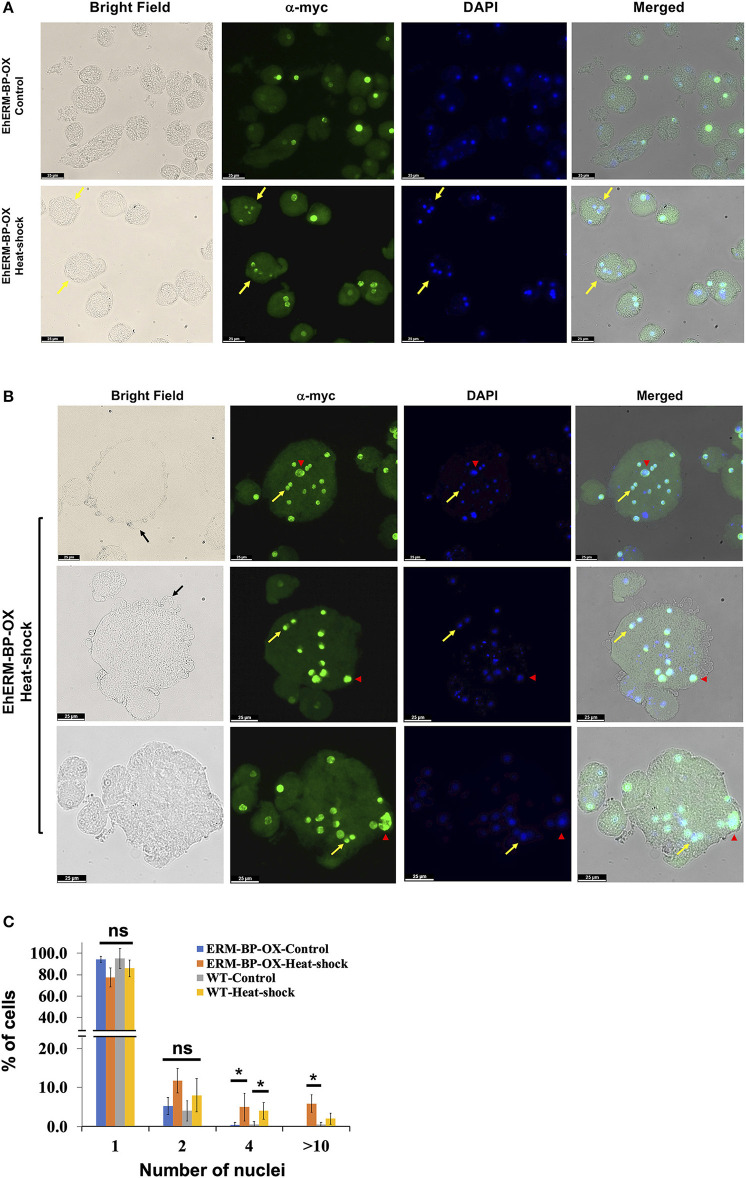
Overexpression of ERM-BP and heat stress induce nuclear division and cell fusion in *E. histolytica*. **(A)** Immunostaining with α-myc antibody in control and heat-shocked ERM-BP-OX cells shown in green. DNA was stained with DAPI (Blue). Yellow arrows indicate the quadri-nucleated cells in heat-shock condition. Scale bars are 25 μm. **(B)** Immunostaining of ERM-BP-OX MGC in heat-shock condition with α-myc antibody shown in green. DNA was stained with DAPI (Blue). Yellow arrow indicates the divided nuclei inside the giant cell and red arrowhead indicates polyploid nuclei. Three fields are shown as representative. Scale bars are 25 μm. **(C)** Percentage of cells with different number of nuclei (1, 2, 4, and >10) in control and heat-shock conditions for both wild type (WT) and ERM-BP-OX are shown. Data are mean ± s.d. (*n* = 2), Student's *t*-test; *P < 0.05. “ns” for not significant.

Overexpression of ERM-BP in *E. invadens* enhanced encystation efficiency and addition of NAD^+^ had an additive effect and further enhanced encystation in ERM-BP-OX cells (Manna et al., 2018). However, when we overexpressed ERM-BP in *E. histolytica* we did not observe calcofluor stained cysts in glucose deprived and osmotic stress condition, even with the addition of excess NAD^+^; instead most of the cells rounded up and died within 16 h of incubation (data not shown). However, upon analyzing *E. histolytica* trophozoites a small percentage of cells with quadrinucleated structures were observed due to heat stress in both WT and ERM-BP overexpressed parasites, suggesting ERM-BP may be transcriptionally active and responsive for the nuclear division in heat stress conditions similar to encystation pathway (Figure 4A). We also observed that in heat-shock conditions and when ERM-BP is overexpressed, cell fusion events occur in *E. histolytica*, leading to the formation of bigger cells with multiple nuclei (Figure 4B). Multiple blebs throughout the cell surface were observed in those big cells, suggesting that these cells are competent for continuous fusion to making even larger cells. To determine whether these giant cells are viable, FDA hydrolysis assay was performed along with staining the cells with cell permeable Hoechst 33342 to stain DNA and revealed that the giant cells are indeed viable (Supplementary Figure 3). Additionally, we analyzed the percentage of ERM-BP-OX cells and wild type cells (WT) containing different numbers of nuclei in control and heat-shock conditions. The data reveals that the number of multi-nucleated cells increase upon heat shock, in both wild-type control cells as well as in ERM-BP overexpressing parasites (Figure 4C).

**Figure 5:**
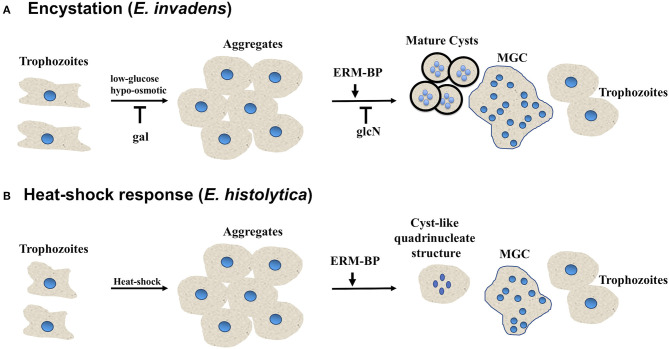
*Entamoeba* encystation and heat-shock response are related. Schematic showing different morphological changes during encystation and heat-shock response in *Entamoeba*. **(A)** *E. invadens* form aggregates in encystation media due to osmotic stress and glucose deprivation. Free galactose (gal) block the multicellular aggregate formation, on the other hand N-acetyl-glucosamine (glcN) does not affect aggregate formation however, it prevents cyst formation. ERM-BP also work downstream of aggregate formation. With the progression of encystation nuclear division take place to make quadrinucleated cyst with thick chitin cyst wall. Cell fusion also take place in encystation conditions, and *Entamoeba* can transform into multinucleated giant cells which are highly motile under confinement may undergo continuous cytofission to generate daughter cells. **(B)** In heat-shock conditions most of the *Entamoeba* become rounded and form aggregates. Nuclear division occurs and quadrinucleated cyst-like structures are observed. Cell fusion also takes place in heat-shock condition and *Entamoeba* can transform into multinucleated giant cells.

Our results suggest that the giant cells are viable, and EhERM-BP may have a functional role in heat-stress response that is similar to its role in encystation. This result supports the notion of previous findings that heat stress and encystation pathway may have some inter-connection with potential overlap in signaling pathways. Overall our results suggest that the encystation pathway and heat-stress response are related in *Entamoeba*. Previous work from other groups and our recent study depicts the model of *Entamoeba* encystation and heat-shock response pathways and summarizes the key factors which involved in both processes (Figure 5). ERM-BP is downstream of cellular aggregation in both encystation and heat-shock response but is upstream of multinucleated giant cell formation and encystation.

## Discussion

The transcription factor ERM-BP plays an important role in regulating encystation in *Entamoeba*. We dissected the domain functions of ERM-BP further by overexpressing the WT and mutant proteins in *Entamoeba*. Overexpression of ERM-BP-DBM and ERM-BP-C198A resulted in mis-localization of the protein in both trophozoites and cysts and significantly reduced encystation efficiency. During encystation, a subset of *E. invadens* parasites also transform into multi-nucleated giant cells (MGC). We demonstrate that silencing of ERM-BP reduced the number and size of giant cells, which contain fewer nuclei. Among different stress responses, heat-stress was reported earlier to have commonalities with encystation in *Entamoeba* (Field et al., 2000). Our present studies support that notion and demonstrated that NAD^+^ levels increase with heat shock, and that overexpression of ERM-BP protects both *E. histolytica* and *E. invadens* parasites against death by heat shock. Furthermore, in *E. histolytica* heat shock results in cell fusion with formation of giant cells, and also nuclear division which produces cyst-like quadrinucleate parasites. We showed that ERM-BP functions downstream of cellular aggregation and is an early regulator of both development and heat shock response in *Entamoeba*. A model that depicts our understanding of ERM-BP in *Entamoeba* development and heat shock is shown in Figure 5.

Previous studies on ERM-BP showed that ERM-BP binds to metabolic cofactor NAD^+^ and this binding facilitates its DNA binding activity to control the expression of cyst-specific genes. Silencing of ERM-BP significantly decreases encystation efficiency and produces ghost like cysts, with defective cyst walls which fail to excyst. *In vitro* studies with recombinant protein from two mutant ERM-BPs identified a DNA binding motif at the N-terminus, in which changing five amino acids (ERM-BP-DBM) affects its DNA binding properties. Additionally, changing a single amino acid (Cys198) in the C-terminus in the nicotinamidase domain (ERM-BP-C198A), affects NAD^+^ binding as well as DNA binding properties. We have now confirmed these findings in parasites with both mutants (ERM-BP-DBM and ERM-BP-C198A) resulting in abnormal cellular localization in both trophozoites and cysts and resulting in significant reduction in formation of viable cysts. In contrast, mutations in ERM-BP-D12A and ERM-BP-K150A did not affect protein localization in trophozoites or cysts. These results recapitulate studies with recombinant protein and ERM-BP function and demonstrate functional consequences in parasites.

Formation of a small numbers of multinucleated giant cells have been observed during encystation of *E. invadens*. In *E. invadens*, these MGCs were formed in multicellular aggregates through the cyto-fusion of multiple trophozoites in the encystation culture media due to osmotic stress and glucose depletion; however, other stress responses, such as heat shock or oxidative stress did not induce MGC formation in *E. invadens* as described earlier (Krishnan and Ghosh, 2018). Cell fusion and formation of MGC is a common feature that develops during various inflammatory reactions, such as infection with tuberculosis, herpes, HIV or other foreign body (Dargent et al., 2000; Brodbeck and Anderson, 2009; McClean and Tobin, 2016). MGCs are special class of giant cell formed by the fusion of monocytes or macrophages and predominantly found in human tissues and are presumed to contribute to the removal of debris from the tissues (Milde et al., 2015; Miron and Bosshardt, 2018). Formation of MGC can be induced *in vitro* in other systems through the use of conditioned medium (Abe et al., 1991), several different cytokines (Most et al., 1997), addition of lectins alone or in combination with interferon (IFN-γ) (Chambers, 1977; Takashima et al., 1993) and addition of antibodies or phorbol myristate acetate (PMA) or a combination of both (Hassan et al., 1989).

In *E. invadens* the molecular triggers which induce MGC formation are not well-understood. The commonalities between the formation of cyst and MGC include the fact that both require cell aggregate formation, and it is plausible that the initial signaling associated with encystation and MGC formation may be same. A number of meiotic genes were reported to be upregulated during encystation (Ehrenkaufer et al., 2013) and quadrinucleate cysts contain smaller haploid nuclei compared to the polyploid trophozoite nucleus. In MGC, nuclei undergo division after cell fusion and after 48 h of encystation mainly smaller haploid nuclei are observed in MGC (Krishnan and Ghosh, 2018), suggesting that meiotic genes might be also active in MGC. Multinucleated cells also observed in *E. histolytica* trophozoites due to delinking of S-phase and cytokinesis (Das and Lohia, 2002)However, there is a distinct difference between MGC and multinucleated *E. histolytica* cells in that the latter are not giant in size as observed in *E. invadens*. Previously, treatment with the Myosin II inhibitor, 2,3-butanedione monoxime (BDM), was observed to inhibit the cytofission without stopping the cell fusion, thus the number of MGCs was not affected by BDM (Krishnan and Ghosh, 2018). These observations indicate the possibility of continuous fusion and cytofission occurring inside the cell aggregates and that the MGCs observed in older encystation cultures could be the final product of such cyclic fusion. Aggregate formation in encystation media is crucial for cyst formation, and multiple cells fused together in this aggregate could lead to the formation of MGCs.

It was previously reported that the heat shock and encystation response in *Entamoeba* are related (Field et al., 2000). Earlier studies revealed the involvement of heat shock protein 90 (HSP90) as a negative regulator of *Entamoeba* encystation (Singh et al., 2015) (43). Messenger RNA for chitinase and Jacob are strongly induced in both encystation and heat stress (Field et al., 2000) and expression of Jacob protein is also evident in the secretory vesicles of heat-shocked *E. invadens*, suggesting an important link between encystation and heat-shock responses (Field et al., 2000). Recent studies demonstrated that Topoisomerase II is highly upregulated in both during encystation and in heat-stress response as well as due to oxidative stress (Varghese and Ghosh, 2020). Our data, that NAD^+^ increases with heat shock, and that overexpression of ERM-BP protects both *E. histolytica* and *E. invadens* parasites against death by heat shock further supports the functional and regulatory link between heat shock and encystation. Furthermore, as we demonstrated that *E. histolytica* heat shock results in cell fusion with giant cells, and also nuclear division which produces cyst-like quadrinucleate parasites it would appear that ERM-BP functions in a parallel manner in both *E. histolytica* and *E. invadens*.

Overall, our work identified that ERM-BP functions downstream of cellular aggregation and is an early regulator of both development and heat shock response in *Entamoeba*. Future studies to dissect the interacting partners of ERM-BP are planned. Definitive proof that ERM-BP regulates development in *E. histolytica* awaits future efforts and development of a system to generate cysts in *E. histolytica*.

## Data Availability Statement

All datasets generated for this study are included in the article/Supplementary Material.

## Author Contributions

DM and DL-A designed and performed the experiments. DM, DL-A, GE, and US analyzed the data and wrote the manuscript. US conceived of the project and aided in manuscript preparation. All authors contributed to the article and approved the submitted version.

## Conflict of Interest

The authors declare that the research was conducted in the absence of any commercial or financial relationships that could be construed as a potential conflict of interest.

## Supplementary Material

Supplementary Figure 1 Transcription factor ERM-BP is upregulated due to heat shock. RT-PCR to detect the expression of *Eh*ERM-BP (EHI_146360) transcript level in *E. histolytica* (HM-1:IMSS) trophozoites in control and heat-stress condition with a loading control (EHI_199600).

Supplementary Figure 2 Viability assay of control and overexpressed-ERM-BP cells from both *E. histolytica* and *E. invadens*. Viability assay was performed by Fluorescein Diacetate (FDA) hydrolysis at different time points of heat stress (0, 1, 2, 3, and 8 h). Data are mean ± s.d. (*n* = 2) Student's *t*-test; ^*^*P* < 0.05.

Supplementary Figure 3 Fluorescein Diacetate (FDA) showing the heat shock induced giant cells are viable. *E. histolytica* cells were heat shocked for 3 h and stained with FDA for viability and Hoechst 33342 to stain DNA. Three fields are shown as representative, yellow arrow indicates the giant cells and red arrowhead showing the dead cells. Scale bars are 25 μm.

Supplementary Movies 1 and 2 *E. invadens* cells were encysted for 72 h and stained with cell permeable Cyto11 (in green to stain DNA) and calcofluor white (in blue to stain cyst wall) in a 96 well plate. Cells were continuously visualized using three channels (Bright Field, FITC, and DAPI) using a Leica CTR6000 microscope and time-lapse images were captured at 1 s intervals. Images were analyzed using Leica LAS-AF software. The movie represents all the channels as merged and 3 frames per second (.mov file).
