# Supplementary material for: The NAD+ Responsive Transcription Factor ERM-BP Functions Downstream of Cellular Aggregation and Is an Early Regulator of Development and Heat Shock Response in Entamoeba
Source: Front Cell Infect Microbiol. 2020 Jul 17;10:363. doi: 10.3389/fcimb.2020.00363 (PMC7379229; doi:10.3389/fcimb.2020.00363)
Supplement: Supplementary Figure 1 — Transcription factor ERM-BP is upregulated due to heat shock. RT-PCR to detect the expression of EhERM-BP (EHI_146360) transcript level in E. histolytica (HM-1:IMSS) trophozoites in control and heat-stress condition with a loading control (EHI_199600). [file Presentation_1.PPTX]

## Slide 1
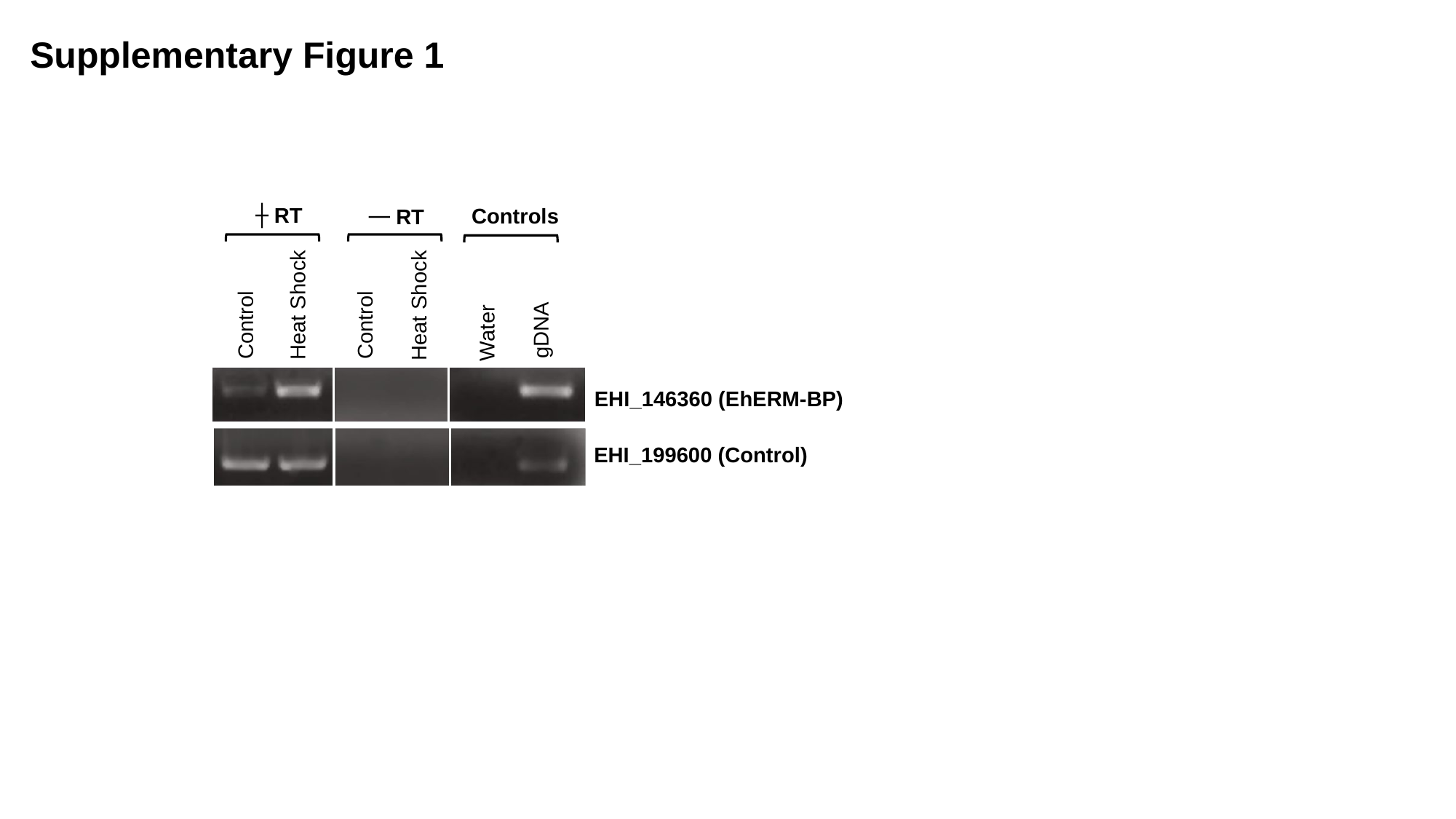

Supplementary Figure 1
┼ RT
Controls
⎯ RT
 Heat Shock
Control
Control
gDNA
Water
EHI_146360 (EhERM-BP)
EHI_199600 (Control)
 Heat Shock

## Slide 2
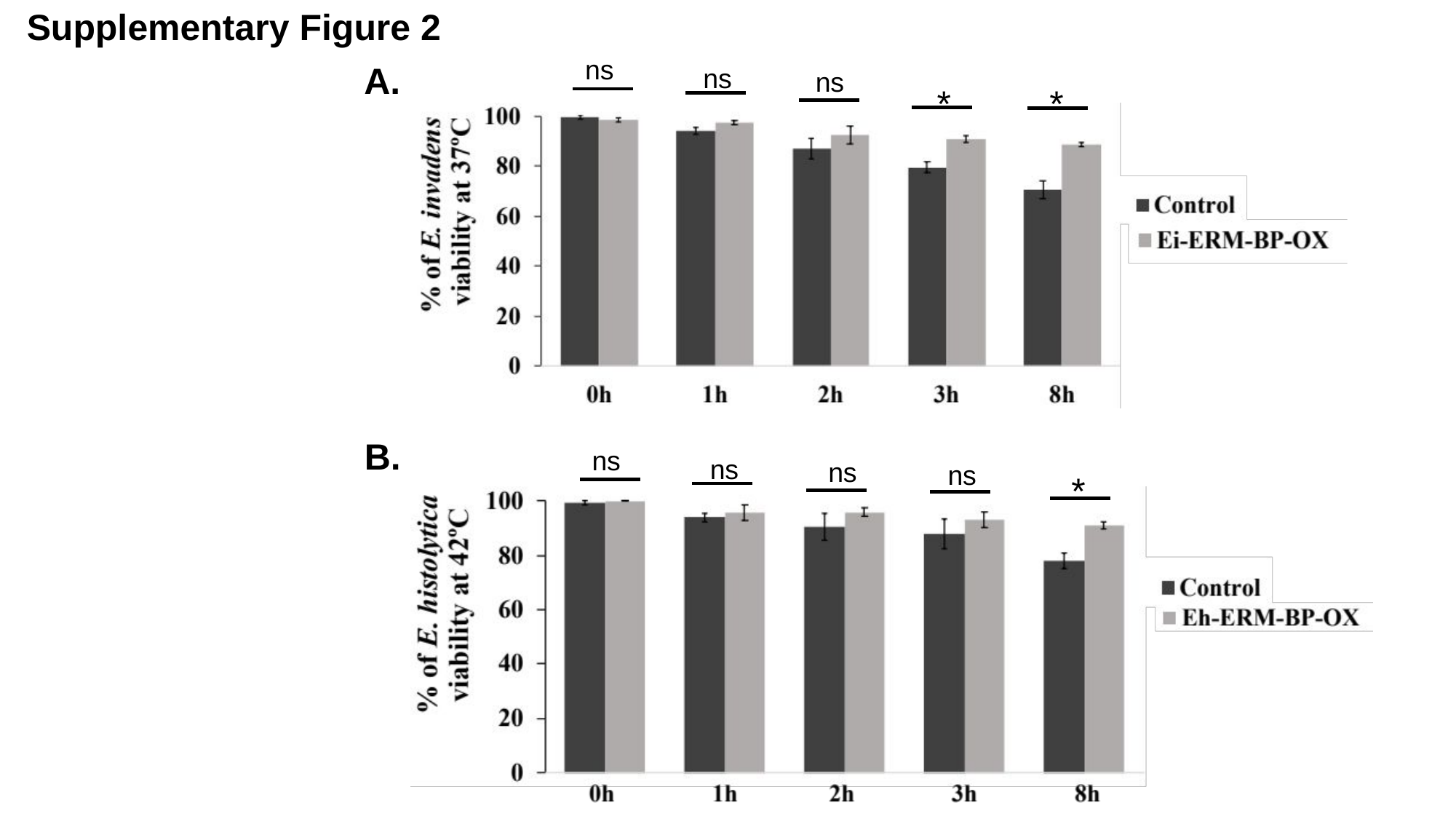

Supplementary Figure 2
ns
ns
ns
*
*
A.
B.
ns
ns
ns
ns
*

## Slide 3
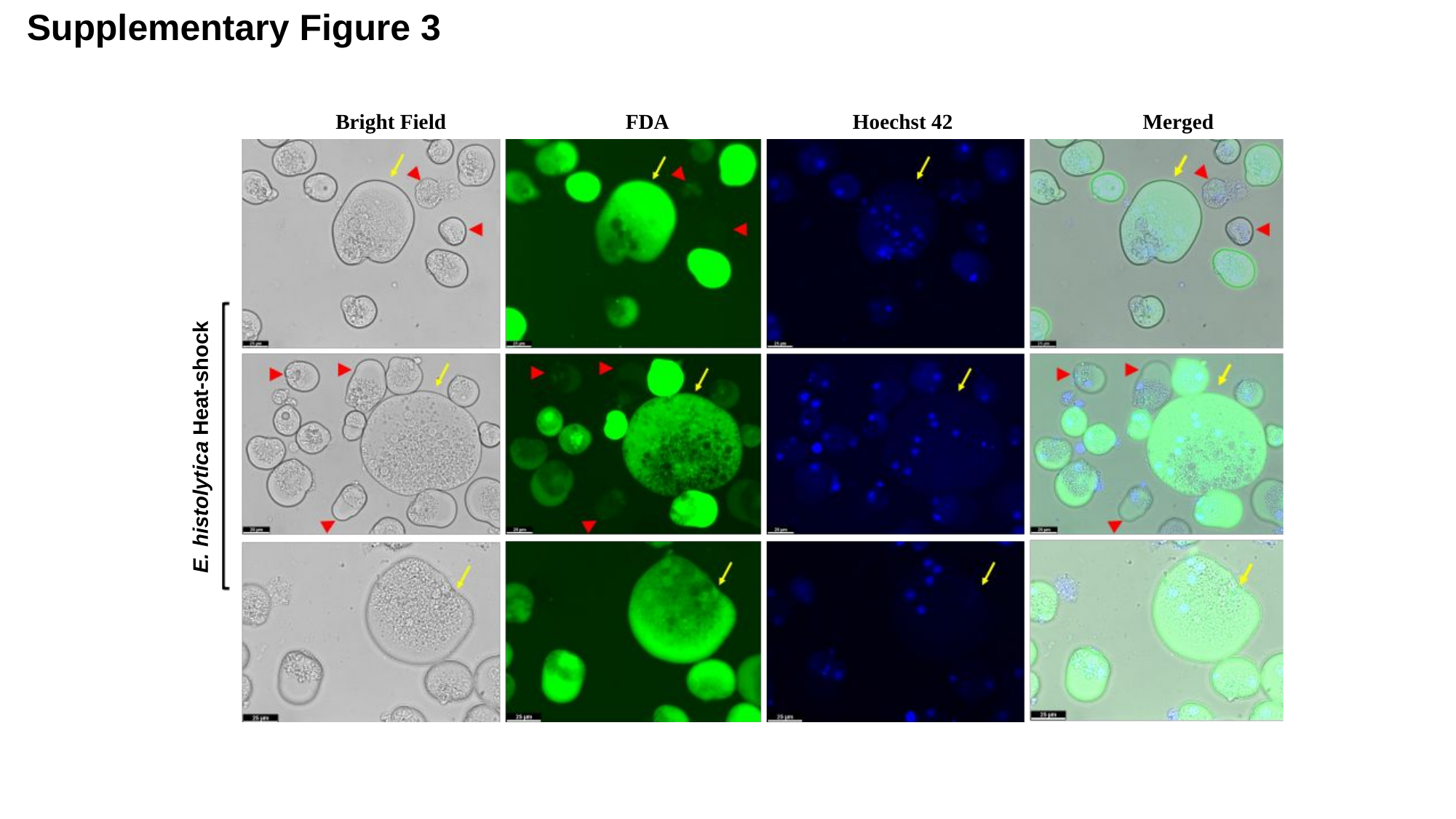

Supplementary Figure 3
Bright Field FDA Hoechst 42 Merged
E. histolytica Heat-shock
